# The Efficacy of Adjunctive Platelet Concentrates in Combined Guided Tissue Regeneration and Bovine Bone Grafting for Intrabony Defects: A Systematic Review

**DOI:** 10.1002/cre2.70421

**Published:** 2026-07-26

**Authors:** Robert Chan, Hugo Lau, Michael Liao, Jiwoo Park, Aaron Ting, Yi Yang Xu, Tulio Fernandez‐Medina

**Affiliations:** ^1^ College of Medicine and Dentistry James Cook University Cairns Australia; ^2^ School of Dentistry The University of Queensland Brisbane Australia

**Keywords:** barrier membrane, bone graft, periodontal regeneration, platelet‐rich

## Abstract

**Objectives:**

Autologous platelet concentrates (APCs) have emerged as biologically active adjuncts in guided tissue regeneration (GTR), aiming to enhance the treatment of intrabony periodontal defects through the local release of growth factors. Despite growing interest, the clinical benefits of combining APCs with bovine‐derived bone grafts (BBG) in the treatment of intrabony periodontal defects remain uncertain. This systematic review aimed to evaluate the clinical efficacy of incorporating APCs alongside GTR and BBG in managing intrabony defects.

**Materials and Methods:**

A comprehensive electronic literature search was conducted across PubMed, Ovid MEDLINE, Scopus, Cochrane, and Web of Science. Inclusion criteria included randomised control trials involving systemically healthy adults with periodontal intrabony defects > 3 mm. Studies were screened based on predefined inclusion and exclusion criteria, and the methodological quality and risk of bias were assessed using the Cochrane Risk of Bias 2.0 tool.

**Results:**

The initial search identified 214 articles, from which eight full‐text studies were screened for eligibility. Five randomised controlled trials met the inclusion criteria. One study reported potential clinical benefits with adjunctive APC use, while the remaining trials showed no statistically significant improvements. All included studies demonstrated concerns regarding risk of bias, primarily due to the absence of predefined protocols, and only one study provided trial outcomes beyond 12 months. Overall, the APC groups exhibited slightly greater mean gains in clinical attachment level (0.1–1.0 mm) and probing pocket depth reduction (0.1–0.8 mm) at 12 months compared with controls.

**Conclusion:**

The adjunctive use of APCs with bovine‐derived bone grafts and guided tissue regeneration did not result in statistically significant improvements in clinical attachment level gain or probing pocket depth reduction. Future studies with standardised methodologies, larger sample sizes, and extended follow‐up durations are needed to clarify the long‐term efficacy of APCs in periodontal regenerative therapy.

## 1 Introduction

Periodontitis is a chronic inflammatory disease characterised by the progressive destruction of tooth‐supporting structures, including the periodontal ligament (PDL) and alveolar bone (Kononen et al. 2019). If left untreated, periodontitis can lead to tooth mobility and ultimately, tooth loss (Kononen et al. 2019). A hallmark feature of Stage III and IV periodontitis is the development of intrabony defects (Kononen et al. 2019). These vertical bone defects are recognised as complexity factors in the current classification of periodontal disease and present significant challenges to successful periodontal therapy (Papapanou et al. 2018; Miron, Moraschini et al. 2025).

In these advanced cases, the primary goal of periodontal therapy is to regenerate the lost PDL, cementum, and alveolar bone (Alqahtani et al. 2023). Among established regenerative strategies, guided tissue regeneration (GTR) has been widely applied clinically (Alqahtani et al. 2023). GTR historically involved the use of barrier membranes (BM) to prevent the rapid migration of epithelial cells and gingival fibroblasts that would otherwise form a long junctional epithelium (Alqahtani et al. 2023). In turn, it creates space to allow slower‐proliferating PDL cells and osteogenic cells to repopulate the defect (Alqahtani et al. 2023). These barrier membranes can be categorised as resorbable, such as collagen membranes, or non‐resorbable, like expanded polytetrafluoroethylene (ePTFE) membranes (Alqahtani et al. 2023; Ferraz 2023). To enhance bone fill and stability, bone graft materials have been incorporated into GTR (Alqahtani et al. 2023; Ferraz 2023). Bone grafting prevents the collapse of the barrier membrane and acts as a scaffold for new bone formation. Autologous bone grafts remain the gold standard for bone grafting, but their use is limited by donor site availability and patient‐related factors (Alqahtani et al. 2023; Ferraz 2023). As a result, bovine‐derived bone grafts (BBG) have become the most commonly used xenografts in dentistry due to their biocompatibility, osteoconductive properties, and structural resemblance to human bone (Alqahtani et al. 2023; Ferraz 2023).

The biological properties of BBG are primarily governed by the particle size and quality of the particulate, specifically ranging between cortical and cancellous granules (Wheeler et al. 1998). The success of GTR hinges on the physicochemical characteristics of BBG, particularly their ion and mineral content, which directly dictate the resulting biological cascade in the microenvironment (Ege et al. 2022). These material properties are crucial because they determine whether the host response results in beneficial angiogenesis, the mineralisation of surrounding undifferentiated tissue, or an adverse foreign body reaction (Rahaman et al. 2025). The most desirable outcome is frustrated phagocytosis, a mechanism where new bone tissue grows directly onto the particle's surface (Wen et al. 2023). This is achieved when the particle prevents the typical healing default of recruiting multinucleated giant cells and forming a dense fibrotic encapsulation mediated by myofibroblasts (Tai et al. 2021). Unfortunately, the crucial surface interface variations that drive these distinct biological responses are frequently overlooked in the existing dental and biomedical literature (Lackington et al. 2022).

Autologous Platelet Concentrates (APCs), such as platelet‐rich plasma (PRP) and platelet‐rich fibrin (PRF), have emerged as valuable biological adjuncts in regenerative periodontal therapy (Al‐Hamed et al. 2019). These products, which are derived from the patient's own venous blood, offer excellent biocompatibility and minimise the risk of adverse outcomes (Al‐Hamed et al. 2019). While both are powerful APCs, their preparation dictates their mechanism of action. PRP is prepared with an anticoagulant, resulting in a liquid form that delivers a rapid “burst” of growth factors upon activation (Ivanovski et al. 2025). In contrast, PRF is produced without an anticoagulant, allowing a natural, gel‐like fibrin matrix to form. This fibrin scaffold effectively traps growth factors and leukocytes, facilitating a slower, more sustained release over time, which is particularly beneficial for long‐term tissue maturation (Ivanovski et al. 2025). In general, APCs enhance wound healing by promoting cellular recruitment, proliferation, and extracellular matrix formation. These mechanisms not only facilitate cell migration but also provide structural support within intrabony defects. Furthermore, APCs aid in blood clot stabilisation and exhibit crucial anti‐inflammatory effects that help modulate the local immune response (Al‐Hamed et al. 2019; Dohan Ehrenfest et al. 2010).

An expanding body of preclinical and in vitro research provides strong support for the comprehensive biological properties of APCs. In vitro, both PRP and PRF release an array of essential growth factors, including platelet‐derived growth factor (PDGF), transforming growth factor‐beta (TGF‐β), vascular endothelial growth factor (VEGF), and insulin‐like growth factor (IGF). These growth factors collectively orchestrate the proliferation, chemotaxis, and osteogenic differentiation of PDL cells and osteoblasts (Fréchette et al. 2005; El‐Sharkawy et al. 2007). Specifically, PRF exudate significantly upregulates proliferation, alkaline phosphatase (ALP) activity, and mineralised nodule formation in human PDL cells in a dose‐dependent manner, alongside the transcriptional upregulation of key osteogenic markers including RUNX2, osteocalcin, and osterix (Li et al. 2018). Similarly, PRP has been shown to stimulate the migration and mitotic activity of both PDL cells and osteoblasts in vitro, with increased responses observed over time and at greater concentrations (Creeper et al. 2009). Furthermore, both preparations exhibit potent angiogenic bioactivity that drives endothelial cell migration and neovascularisation (Kobayashi et al. 2015). It is particularly noteworthy that PRF membranes exhibit prolonged angiogenic kinetics in standardised in vitro chorioallantoic membrane (CAM) assays (Kobayashi et al. 2015). In vivo, PRF combined with PDL stem cells yields significantly greater new bone formation in rodent models, as confirmed by micro‐computed tomography (µ‐CT) and histomorphometric analysis (Duan et al. 2018). Moreover, the application of autologous PRF in a canine split‐mouth periodontal model markedly improved clinical parameters, suppressed pro‐inflammatory cytokine expression, and enhanced both collagen deposition and growth factor gene expression (Kornsuthisopon et al. 2020).

While these preclinical insights establish a compelling foundation for utilising APCs as powerful adjuncts within GTR protocols, translated clinical evidence remains equivocal. Recent systematic reviews and meta‐analyses have largely indicated that the adjunctive use of APCs—including PRP or PRFs—with bone grafts (BGs) may enhance outcomes like Probing Pocket Depth (PPD) reduction and Clinical Attachment Level (CAL) gain, compared to using bone grafts alone (Miron, Moraschini et al. 2025; Panda et al. 2016; Silva et al. 2024). In vivo findings on specific APCs in human studies, however, are mixed: some reports suggest PRF offers greater improvement alongside guided tissue regeneration (GTR), while others find limited evidence or suggest PRP combined with BGs alone provides a significant effect (Miron, Moraschini et al. 2025; Panda et al. 2016; Silva et al. 2024). These studies hint at a potential difference in the therapeutic power of PRP versus PRF (Panda et al. 2016). Critically, no current systematic review has comprehensively evaluated the specific triple combination of APCs (PRF or PRP), BBG, and GTR (Miron, Moraschini et al. 2025). Furthermore, the existing literature often lacks specificity regarding factors that strongly influence regenerative outcomes, such as the type of barrier membranes and the precise characteristics (nature, composition, and particle size) of the bone grafts used (Miron, Moraschini et al. 2025).

To address these distinct literature gaps, this systematic review aims to provide a rigorous, comprehensive evaluation of this triple therapeutic approach. Specifically, it evaluates the comparative impact of adjunctive APCs on CAL gain and PPD reduction when strictly combined with BBG and GTR for the treatment of intrabony periodontal defects. By systematically accounting for the critical confounding biomaterial variables, such as membrane types and graft characteristics, this review seeks to establish a clearer benchmark for clinical predictability and therapeutic efficacy in regenerative periodontal therapies.

## 2 Methods

### 2.1 Research Protocol and Eligibility Criteria

A review protocol was developed and registered with *PROSPERO (CRD420251022525)* before data extraction and risk of bias assessment. All authors participated in the development of the protocol, including the research question and the inclusion and exclusion criteria, which were written up for each element of the *PICO model (population, intervention, control, outcome)* (Eriksen and Frandsen 2018). Eligibility criteria included systemically healthy patients with periodontal intrabony defects > 3 mm in depth. Studies containing adolescent (< 18 years old) and elderly (> 80 years old) participants were excluded. Accepted study designs included randomised control trials (RCTs) and pilot RCTs published between January 1, 2002, and July 30, 2025. The inclusion criteria for the intervention included GTR, particularly the use of a bovine graft and a membrane, alongside the application of an APC such as PRF or PRP. Studies reporting the use of GTR without an additional APC element as a control were included. Lastly, CAL gain was decided as the primary outcome and served as the inclusion criterion for the articles.

### 2.2 Study Selection Process

The literature search was conducted across five major databases: Scopus, Web of Science, Cochrane Central, PubMed, and Medline (OVID). A combination of Medical Subject Headings (MeSH) and keywords were used to formulate the search strategy, including terms such as “autologous platelet concentrate*” OR “APC” OR “PRP” OR “PRF” OR “platelet rich”; Bovine OR “Bio‐Oss”; “bone graft” OR “guided tissue regeneration” OR “GTR”; and “intrabony defect*” OR “intra‐bony” OR “intrabony*” (Appendix Table S1). Automated filters were applied where available to limit results to RCTs. All search results between January 1, 2002, and July 30, 2025, were imported (EndNote 2025, Philadelphia‐US) for comprehensive reference management and the removal of duplicate entries. Subsequently, two independent researchers (A.T. and Y.X.) screened the titles and abstracts of each article against a predefined set of inclusion and exclusion criteria. Full‐text evaluation of selected studies was performed by the same two researchers. Any discrepancies were resolved by a third independent reviewer (H.L.). Inter‐rater reliability was assessed using Cohen's Kappa statistic at the full‐text screening stage, with the results indicative of perfect agreement (Appendix Table S1) (McHugh 2012).

### 2.3 Data Points Extracted

The data points extracted included study design, population (e.g., type of periodontal intra‐bony defect), intervention and control variables (e.g., bovine graft particle sizes and membrane type), study outcomes (e.g., CAL and PPD), and duration of the follow‐up period. Missing data were recorded, and discrepancies between article results were noted. The available results were standardised by calculating the uncertainties where possible, and full working out is presented in Table 1.

**Table 1:** Characteristics and outcomes of the included studies.

Study characteristics								Study results						APC method preparation
Author (year)	Study design; follow‐up	No. of participants; gender; age range	Intervention group	Control group	Bovine graft particle size (mm)	Smoker (No, Yes)	Bone defect type	Mean CAL gain over 6 months (mm)	Mean CAL gain over 12 months (mm)	Mean CAL gain over 24 months (mm)	Mean PPD reduction over 6 months (mm)	Mean PPD reduction over 12 months (mm)	Mean PPD reduction over 24 months (mm)	Centrifugation system; volume of blood drawn; centrifugation parameters speed (rpm) × time (min)
Döri et al. (2007a)	RCT; 1 year	30; 16 females, 14 males; 28–56 years	BioOSS granules†/PRP/Bio‐Gide collagen membrane	Bio‐OSS granules†/Bio‐Gide collagen membrane	0.25–1.0	No	Mainly 1–2 walled defects. 1–2 wall: *n* = 9 (intervention), *n* = 10 (control) 2 wall: *n* = 4 (intervention), *n* = 3 (control) 3 wall: *n* = 2 (intervention), *n* = 2 (control)	NR	NBM/PRP/GTR: 4.5 ± 1.1* NBM/GTR: 4.6 ± 1.1	N/A	NR	NBM/PRP/GTR: 5.5 ± 1.3* NBM/GTR: 5.5 ± 1.7	N/A	The Curasan kit; 8.5 mL; first spin 2400 rpm for 10 min; second spin 3600 rpm for 15 min
Döri et al. (2007b)	RCT; 1 year	24; 14 female, 10 male; 26–55 years	PRP/BioOSS†/ePTFE membrane	Bio‐OSS†/ePTFE membrane	0.25–1.0	No	Mixed of 1–2 walled defects. Intervention (*n* = 12): 1–2 wall (*n* = 10), 2 wall (*n* = 2) control (*n* = 12): 1–2 wall (*n* = 9), 2 wall (*n* = 3)	NR	PRP/Bio‐OSS/GTR: 4.7 ± 1.1* ABBM/GTR: 4.6 ± 0.8	N/A	NR	PRP/Bio‐OSS/GTR: 5.5 ± 1.2* ABBM/GTR: 5.7 ± 1.2	N/A	The Curasan kit; 8.0 mL; first spin 2400 rpm for 10 min; second spin 3600 rpm for 15 min
Dőri et al. (2022)	RCT; 6 months	34; NR; NR	NBM†/PRP/ePTFE membrane	NBM†/ePTFE membrane	NR	No	Exhibited at least one deep intrabony defect (probing depth, PD: 8–10 mm) Type of defect = NR	NBM/PRP/GTR: (12.3 ± 2.4) − (4.8 ± 1.8) = 7.5 ± 3.0* NBM/GTR: (9.7 ± 2.2) − (4.5 ± 2.0) = 5.2 ± 3.0*	N/A	N/A	N/A	N/A	N/A	The Curasan kit; NR; NR
Camargo et al. (2009)	RCT; 6 months	23; 14 females and 9 males; 34–67 years	PRP/BPBM†/Collagen membrane	BPBM†/Collagen membrane	0.25–1.0	11 smokers and 12 non‐smokers	Intervention: 19 2‐wall defects and four 3‐wall defects control: 17 2‐wall defects and six 3‐wall defects.	PRP/BPBM/GTR: Buccal: 4.38 ± 1.22 Lingual: 4.12 ± 1.18 BPBM/GTR: Buccal: 3.56 ± 1.21 Lingual: 3.34 ± 1.18	N/A	N/A	PRP/BPBM/GTR: Buccal: 4.88 ± 1.08 Lingual: 4.72 ± 1.12 BPBM/GTR: Buccal: 4.16 ± 1.11 Lingual: 3.82 ± 0.98	N/A	N/A	The Curasan kit; 10 mL; 5,600 rpm for 6 min
Liu et al. (2021)	RCT; 6 months, 1 year, and 2 years	14; 11 females and 4 males; 24–60 years	BPBM†/PRF/Collagen membrane	BPBM†/Collagen membrane	NR	No	If any patient had more than one defect on one side, only the worst defect on each side was analyzed. Type of defect = NR	BPBM/PRF/GTR: Buccal: 2.9 ± 0.4** Lingual: 3.0 ± 0.9 BPBM/GTR: Buccal: 2.0 ± 0.9 Lingual: 2.6 ± 0.6	BPBM/PRF/GTR: Buccal: 3.2 ± 0.6** Lingual: 3.1 ± 0.8** BPBM/GTR: Buccal: 2.1 ± 1.1 Lingual: 2.1 ± 0.7	BPBM/PRF/GTR: Buccal: 3.1 ± 0.5** Lingual: 3.1 ± 0.7** BPBM/GTR: Buccal: 2.1 ± 1.1 Lingual: 2.0 ± 0.8	BPBM/PRF/GTR: Buccal: (4.6 ± 1.2) − (1.9 ± 1.0) = 2.7 ± 1.6 Lingual: (6.0 ± 0.9) − (2.6 ± 0.9) = 3.4 ± 1.3 BPBM/GTR: Buccal: (4.8 ± 1.5) − (2.4 ± 0.6) = 2.4 ± 1.6 Lingual: (6.0 ± 0.9) − (3.0 ± 0.7) = 3.0 ± 1.1	BPBM/PRF/GTR: Buccal: (4.6 ± 1.2) − (1.9 ± 0.7) = 2.7 ± 1.4 Lingual: (6.0 ± 0.9) − (2.6 ± 0.8) = 3.4 ± 1.2** BPBM/GTR: Buccal: (4.8 ± 1.5) − (2.4 ± 0.8) = 2.4 ± 1.7 Lingual: (6.0 ± 0.9) − (3.2 ± 0.7) = 2.8 ± 1.1	BPBM/PRF/GTR: Buccal: (4.6 ± 1.2) − (1.9 ± 0.5) = 2.7 ± 1.3** Lingual: (6.0 ± 0.9) − (2.7 ± 0.6) = 3.3 ± 1.1** BPBM/GTR: Buccal: (4.8 ± 1.5) − (2.4 ± 0.6) = 2.4 ± 1.6 Lingual: (6.0 ± 0.9) − (3.4 ± 0.8) = 2.6 ± 1.2 (Kononen et al. 2019)	Anke, TDL80‐2B; 10 mL; 700 rpm for 3 min

Abbreviations: BPBM = bovine porous bone mineral, ePTFE = expanded polytetrafluoroethylene membrane, N/A = not applicable, NBM = natural bone mineral, NR = not reported, PRP = platelet‐rich plasma, rpm = rotations per minute.

* *p* < 0.001

** *p* < 0.05.

^†^ Cancellous granules (bone quality).

### 2.4 Risk of Bias Assessment

The risk of bias in the included studies was independently assessed by two reviewers (J.P. and R.C.) using the Cochrane Risk of Bias 2.0 (RoB 2.0) tool (Sterne et al. 2019). Disagreements between the two reviewers were resolved by a third reviewer (M.L.). This tool evaluates five key domains: (i) bias from the randomisation process; (ii) deviations from intended interventions; (iii) missing outcome data; (iv) measurement of the outcome; and (v) the selection of the reported result. A flowchart‐based system then guides a judgment for each domain as “low risk,” “some concerns,” or “high risk,” leading to an overall risk of bias judgment for each study (Sterne et al. 2019).

## 3 Results

### 3.1 Study Selection

**Figure 1:**
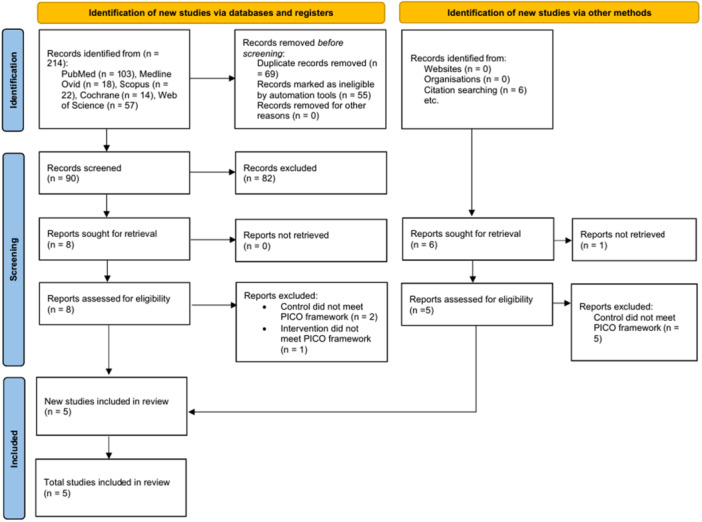
Preferred Reporting Items for Systematic Reviews and Meta‐Analyses (PRISMA) flowchart of the study selection process. *n* = number, PICO = population intervention comparison and outcome.

The initial electronic database search yielded 214 records, distributed as follows: PubMed (*n* = 103), Medline Ovid (*n* = 18), Scopus (*n* = 22), Cochrane (*n* = 14), and Web of Science (*n* = 57). Following the removal of duplicates, 90 unique studies proceeded to title and abstract screening, which resulted in the exclusion of 82 articles. Consequently, eight full‐text articles were retrieved and subjected to detailed analysis, of which three were ultimately excluded. The primary reasons for exclusion at this stage included non‐adherence to the predefined PICO framework (e.g., irrelevant or missing intervention variables), inappropriate study designs, or full‐text inaccessibility (Figure 1).

An additional six records, identified through citation searching, were assessed for eligibility. Of these, five were retrieved, and all were subsequently excluded as they did not meet the PICO framework's control criteria. Ultimately, the systematic review was conducted on a final corpus of five studies.

### 3.2 Study Characteristics

The details regarding study characteristics are presented in Table 1. A total of 125 patients were included across the five included studies. Two studies employed a split‐mouth design, involving 37 patients (Camargo et al. 2009; Liu et al. 2021), while the remaining three studies used a parallel‐group design with 88 total patients (Döri et al. 2007a, 2007b). Noticeably, three studies were authored by Döri et al. (2007a, 2007b, 2022) with two (both published in 2007) sharing an identical author team (Döri et al. 2007a, 2007b). All five studies evaluated the effect of using bovine bone substitutes—specifically Bio‐OSS (generic name is deproteinised bovine bone mineral), bovine porous bone mineral (BPBM)—with or without APCs and a BM (collagen or ePTFE) for treating intrabony periodontal defects. While the APCs comprised PRP in four studies (Camargo et al. 2009; Döri et al. 2007a, 2007b, 2022) and PRF in one (Liu et al. 2021), methodological inconsistencies were evident across the cohort. Furthermore, centrifugation systems and protocols varied significantly, with four studies (Camargo et al. 2009; Döri et al. 2007a) using the Curasan kit (80%) and the remaining studies employing a variety of methods or failing to report the protocol. Notably, one study enrolled smokers who were still considered “systemically healthy” (Camargo et al. 2009) and follow‐up periods ranged from 6 to 24 months, with detailed data provided in Table 1.

### 3.3 Overall Risk of Bias

All five included studies were assessed as having low risk of bias in four domains and some concerns in one domain. The concerns primarily relate to bias in the selection of the reported result, as the studies did not pre‐specify which statistical outcomes would be reported in advance. Table 2 demonstrates the five domains evaluated for each study.

**Table 2:** Risk of bias domains.

		Risk of bias domains					
		D1	D2	D3	D4	D5	Overall
Study	Döri et al. (2007a)	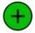	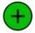	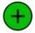	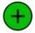	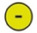	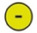
	Döri et al. (2007b)	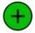	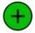	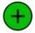	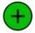	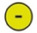	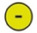
	Camargo et al. (2009)	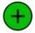	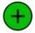	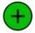	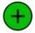	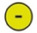	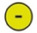
	Liu et al. (2021)	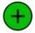	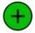	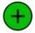	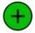	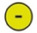	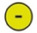
	Döri et al. (2022)	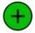	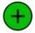	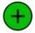	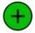	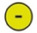	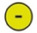
		Domains: D1: Bias arising from the randomisation process. D2: Bias due to deviations from the intended intervention. D3: Bias due to missing outcome data. D4: Bias in measurement of the outcome. D5: Bias in the selection of the reported result.				Judgment 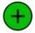 Low 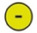 Some concerns	

### 3.4 Synthesis of Results

Across follow‐up periods, both APC/BBG/GTR and BBG/GTR alone demonstrated improvements in CAL gain and PPD reduction (Table 1). Most studies showed slightly greater CAL gains and PPD reductions in the APC groups. For the only study that reported a 24‐month follow‐up period, minimal differences between 12 and 24 months were shown in both control and intervention groups. It should be noted that of the five included studies, only Liu et al. (2021) examined PRF, while the others evaluated PRP.

**Figure 2:**
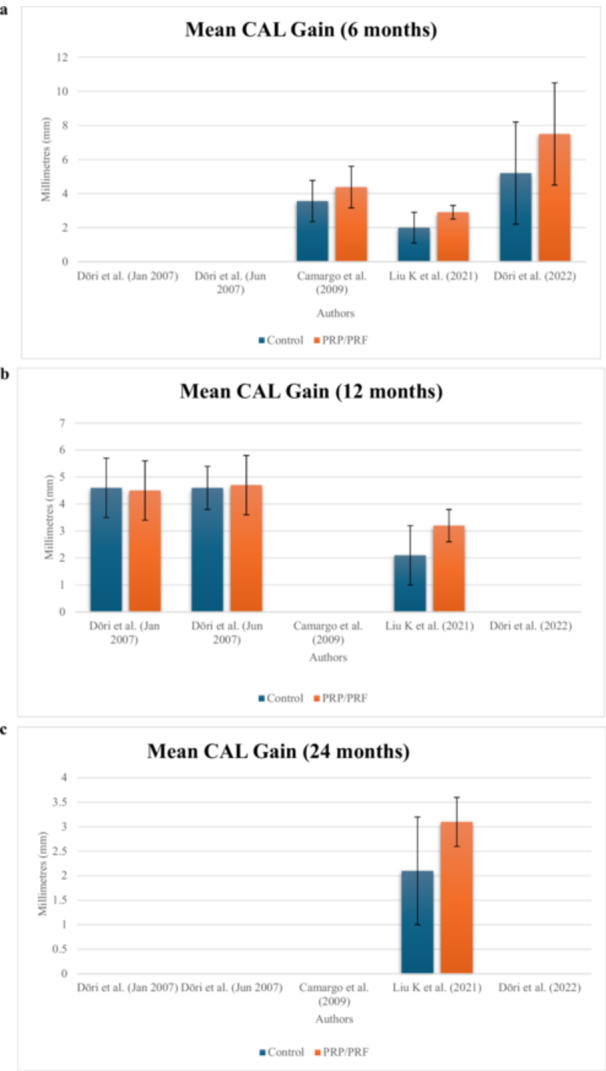
(a) Primary outcome: mean CAL gain at 6 months, (b) primary outcome: mean CAL gain at 12 months, and (c) primary outcome: mean CAL gain at 24 months.

Data from this review on CAL gain and PPD reduction indicate inconsistent outcomes when APCs are used as an adjunct to BBG and GTR. At the 6‐month mark, limited data from three studies showed that CAL gains ranged from approximately 2.0 to 3.5 mm in control groups (BBG/GTR) and 2.9 to 4.5 mm in the APC/BBG/GTR groups (Figure 2a). By 12 months, three studies demonstrated slightly greater CAL gains in the APC groups, with differences of about 0.1–1.0 mm (Figure 2b). One study at 24 months reported sustained CAL gains in both groups, with a small difference favoring the APC group (Figure 2c).

Similarly, for PPD reduction, only two studies had limited data at 6 months, showing gains of 2.4–4.2 mm for control groups and 2.7–4.9 mm for APC groups (Supporting Information S1: Appendix C1). By 12 months, three studies reported slightly greater PPD reduction in the APC groups (0.1–0.8 mm difference) (Supporting Information: S1 Appendix C2). At 24 months, only one study was available, which showed a PPD reduction favoring the APC group (Supporting Information S1: Appendix C3). Overall, while some limited data suggest a slight trend toward better outcomes in the APC groups, the evidence is insufficient to conclude that adding APCs provides a statistically significant clinical advantage.

**Figure 3:**
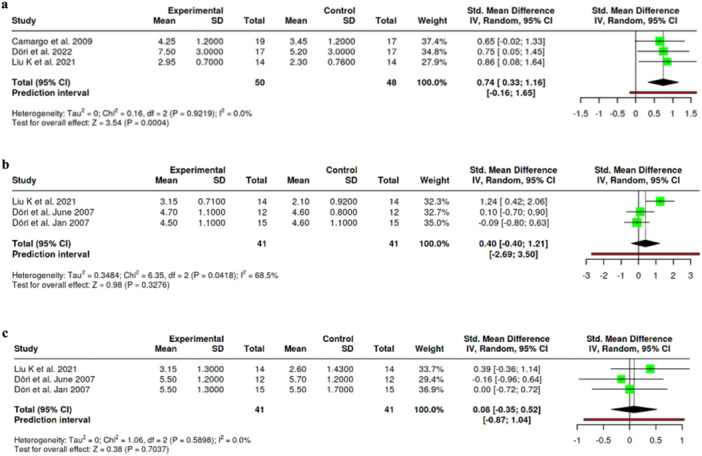
(a) Mean CAL gain over 6 months, (b) mean CAL gain over 12 months, and (c) mean PPD reduction over 12 months.

The pooled effects of APCs on CAL gain and PPD reduction are illustrated in Figure 3. At 6 months, adjunctive APC use resulted in a significantly greater CAL gain compared with BBG + GTR alone (STD = 0.74, 95% CI: 0.33–1.16) (Figure 3a). At 12 months, the pooled estimate for CAL gain showed no significant differences between groups and showed substantial heterogeneity (STD = 0.40, 95% CI: −0.40 to 1.21, *I* ^2^ = 68.5%) (Figure 3b). Further, the PPD reduction at 12 months demonstrated no significant benefit of APCs over controls (STD = 0.08, 95% CI: −0.35 to 0.52) (Figure 3c). These findings suggest that any early advantage associated with APCs may diminish over time and should be interpreted cautiously, given the limited number of available studies.

## 4 Discussion

This systematic review's primary objective was to evaluate the clinical outcomes of combining BBG and GTR with or without the adjunctive use of APCs for treating intrabony periodontal defects. The collective findings suggest a notable lack of robust evidence to support significant clinical advantages from adding APCs. The current study is the first systematic review to evaluate APC/BBG/GTR compared to BBG/GTR. Consequently, a direct comparison of the present findings in the context of the existing literature is not feasible. However, several recent systematic reviews and meta‐analyses have investigated the therapeutic potential of APCs as adjuncts to similar regenerative therapies, including their use in combination with open flap debridement (OFD), for the management of periodontal intrabony defects. The results of the present systematic review regarding the adjunctive use of APCs with GTR and BBG corroborate the conclusions drawn from these similar studies.

Conversely, only one RCT, conducted by Liu et al. (2021) concluded that adding PRF is clinically more effective and provides better outcomes compared to using BBG and GTR alone. However, the authors emphasised that these results are preliminary and require further investigation. While studies by Döri et al. (2007a, 2007b, 2022) and Camargo et al. (2009) suggested potential benefits of PRP, the current review found that the clinical evidence indicates PRP provides only limited and inconsistent benefits when used with BBG and GTR. In contrast, the study by Liu et al. (2021) revealed that PRF demonstrated more reliable improvements in CAL gain and PPD reduction, suggesting it may be superior to PRP in predictability and effectiveness for periodontal regeneration. Döri et al. (2022) found that improvements from PRP application were small and insignificant. They also determined that PRP had no particular effect on the integration quality of ePTFE membranes when used in combination with BBG, concluding that both methods—with and without APCs—were equally effective for treating intrabony periodontal defects.

Regarding membrane type, no significant differences in outcomes were reported between ePTFE and collagen membranes. In a separate study, Döri et al. (2007a, 2007b, 2022) tested the adjunctive use of APCs with both ePTFE and collagen membranes, demonstrating no significant advantage associated with either membrane type.

Nevertheless, Miron, Moraschini et al. (2025) reported that adding PRF to BG/BM/OFD did not result in greater CAL gain or PPD reduction compared to BG/BM/OFD alone. Similarly, Panda et al. (2016) found that the addition of PRP to GTR and BG did not produce statistically significant improvements in either CAL gain or PPD reduction, suggesting that any potential effect of PRP may have been masked by the regenerative impact of GTR. Silva et al. (2024) also reported comparable findings, with PRF combined with BG showing no significant advantage over BG alone in terms of CAL gain or PPD reduction. Furthermore, when comparing PRP versus PRF, the findings of this systematic review support Miron, Moraschini et al. (2025), where PRF/OFD produced similar results to PRP/OFD. However, Panda et al. (2016) evaluated PRP/OFD and PRF/OFD separately rather than directly comparing them. They found that the single PRP/OFD study did not yield significant improvements in CAL gain or PPD reduction compared with OFD alone, whereas two of the four PRF/OFD studies showed significant gains. This suggests that, while Miron, Moraschini et al. (2025) concluded equivalence between PRP and PRF, the individual study outcomes in Panda et al. (2016) indicate that PRF may offer additional benefits in some cases, possibly due to its fibrin architecture enhancing wound stability and healing. Discrepancies in these results may be due to heterogeneity in study designs, surgical techniques, follow‐up duration, and platelet concentrate preparation methods, as acknowledged by Panda et al. (2016). To isolate the distinct therapeutic effects of PRP and PRF, prospective RCTs must evaluate both modalities within a strictly controlled BBG/GTR protocol, utilising standardised baseline preparation frameworks and synchronised follow‐up intervals.

The observed differences in clinical outcomes between PRP and PRF are likely attributable to their distinct biological properties. PRP is prepared from anticoagulated whole blood and, upon activation with thrombin or calcium chloride, releases a rapid bolus of growth factors; however, its liquid formulation renders it susceptible to washout from the surgical site, limiting sustained bioavailability. In contrast, PRF is produced without anticoagulants, forming a three‐dimensional fibrin scaffold that entraps platelets, leukocytes, and growth factors (Bielecki and Dohan Ehrenfest 2012), facilitating a slower, sustained release of bioactive molecules including VEGF and PDGF (Kobayashi et al. 2015; Khamis et al. 2025). Its membrane form can also be trimmed and adapted to the defect geometry in ways that a liquid PRP preparation cannot. A comparative RCT by Pradeep et al. (2012) directly evaluating PRF versus PRP in three‐wall intrabony defects found that PRF demonstrated greater mean bone fill, suggesting a clinical advantage attributable to its scaffold properties and sustained growth factor release. A comprehensive review by (Miron, Estrin et al. (2025) encompassing 30 years of APC research, similarly concluded that PRF tends to produce more consistent regenerative outcomes than PRP across multiple clinical indications. Meta‐analytic evidence has further confirmed the adjunctive benefit of leukocyte‐ and platelet‐rich fibrin (L‐PRF) for PPD reduction, CAL gain, and radiographic defect improvement in periodontal bone defects (Pepelassi and Deligianni 2022). Subsequent research should elucidate the comparative efficacy of distinct PRF sub‐formulations—specifically L‐PRF, advanced PRF (A‐PRF), and injectable PRF (i‐PRF)—within the definitive parameters of a BBG/GTR protocol. Crucially, prospective clinical data should be stratified not only by osseous defect architecture but also by patient‐specific variables, including systemic comorbidities and tobacco consumption profiles.

All included studies shared notable methodological and reporting strengths. Each study was an RCT with two studies adopting a split‐design (Camargo et al. 2009; Liu et al. 2021), reducing the selection bias and inter‐subject variability. Additionally, they consistently measured objective clinical outcomes such as CAL gain and PPD reduction, with examiner calibration enhancing reliability. The results were presented with clarity, using appropriate statistical analyses and defined significance levels, while follow‐up intervals allowed both short‐ and long‐term assessment of outcomes. That said, only one study reported results for all three follow‐up periods of 6, 12, and 24 months (Liu et al. 2021). Importantly, all studies confirmed no baseline differences between groups, further strengthening the validity of their comparisons (Liu et al. 2021).

Substantial heterogeneity was observed across trials in the protocols used for APC preparation. Differences between studies were primarily observed in the volume of blood used, centrifugation speed, and duration, while one study (Dőri et al. 2022) reported incomplete information regarding their preparation methods. That compromises the comparability of outcomes, as even minor deviations in protocol can significantly influence platelet yield and the biological quality of the final product. A further concern was that two studies did not assess baseline donor platelet counts (Döri et al. 2007b, 2022). The Food and Drug Administration recommends a minimum donor platelet count to ensure adequate preparation quality (Rahman et al. 2024). Failure to document this standard introduces the risk of suboptimal preparations and may undermine the validity of results. Moreover, several trials were authored by the same research group, raising the possibility of overlapping patient cohorts and the associated risk of bias (Liu et al. 2021; Dőri et al. 2022).

Two studies did not report graft particle sizes (Liu et al. 2021; Dőri et al. 2022). Graft particles function as scaffolds that facilitate bone formation, with smaller particles providing enhanced cellular infiltration and vascularisation, making them more predictable for regeneration than larger particles. Similarly, Liu et al. (2021) omitted information regarding the pore size of the graft material, a parameter fundamental to bone regeneration as it governs the space available for cellular migration (Zhou et al. 2011). Furthermore, two studies failed to specify the number of residual intrabony walls, while others included a mix of one‐, two‐, and three‐walled defects (Liu et al. 2021; Dőri et al. 2022). Since defect morphology strongly influences regenerative potential, with multi‐walled defects generally exhibiting greater CAL gains, the omission of such information limits the comparability and interpretation of reported outcomes across trials (Nibali et al. 2021). Therefore, prospective research should implement outcome stratification based on vertical defect anatomy and provide exhaustive reporting of all bone graft parameters, in strict compliance with the evidence‐based CONSORT framework for surgical trials (Merkow et al. 2021).

A critical and frequently underreported source of variability in PRF research relates to centrifugation protocol and collection tube type. As highlighted by Miron et al. (2019), many studies report only RPM without specifying the relative centrifugal force (RCF) at the clot position—a parameter that cannot be meaningfully compared across centrifuges with differing rotor geometries, rendering reliable replication essentially impossible. Low‐speed centrifugation protocols (e.g., approximately 700 rpm for i‐PRF) produce biologically distinct products compared with conventional L‐PRF protocols (e.g. approximately 2700 rpm), with notable differences in clot size, cellular content, and growth factor entrapment (Alghofaily et al. 2024). The composition of the collection tube constitutes a further confounding variable: silica‐coated tubes may introduce particle contamination that alters the fibrin matrix and biological properties of the PRF product (Gummaluri et al. 2023), whereas titanium tubes have been proposed as an inert alternative, resulting in the introduction of titanium‐PRF (T‐PRF). Scanning electron microscopy and histomorphometric analyses have demonstrated that T‐PRF yields a denser, more highly organised fibrin network with a larger fibrin‐covered surface area compared with conventional glass/silica‐prepared PRF, an effect attributed to the differential platelet activation kinetics at the titanium–blood interface (Tunali et al. 2014). Clinically, this more polymerised fibrin architecture has been associated with elevated early growth factor concentrations in the gingival crevicular fluid and improved periodontal regenerative outcomes in randomised split‐mouth studies (Arabaci and Albayrak 2018; Ozkal Eminoglu et al. 2024). These findings highlight the influence of PRF preparation protocols, including collection tube chemistry, on reported clinical outcomes. Therefore, authors should systematically disclose essential technical parameters, including the localised RCF at the specimen site, spin time, rotor radius and angulation, and tube chemistry. Standardising these parameters is critical for facilitating meaningful comparative analyses and cross‐study synthesis, as clarity and uniformity in future literature depend on adherence to validated reporting guidelines (Miron et al. 2019; Kobayashi et al. 2012).

Despite long‐term studies reporting minimal overall clinical differences, variation in membrane type may still confound outcome interpretation, since their distinct biological and mechanical properties critically influence tissue healing (Kunrath et al. 2025). Non‐resorbable membranes, such as e‐PTFE, provide superior rigidity and space maintenance, preventing soft tissue collapse into the defect and thereby enhancing osteo‐conduction (Ayari 2022). However, their higher exposure and infection risk can compromise their efficacy (Ayari 2022). Conversely, resorbable collagen membranes promote angiogenesis and cellular migration, closely mimicking periodontal connective tissue, but their limited mechanical strength and rapid or unpredictable degradation can lead to premature collapse (Ayari 2022). These functional differences underscore the importance of consistent membrane selection across studies to enable meaningful comparison of regenerative outcomes.

This systematic review's limitations primarily stem from its narrow search criteria, which yielded a small number of eligible studies and consequently limited the overall strength of the evidence. Furthermore, while a risk of bias assessment was performed, its reliability was compromised by the inability to access missing study protocols, which may have obscured unreported methodological deviations. Given the uncertain clinical benefits and high costs of using APCs as an adjunctive therapy, clinicians should approach their application with caution until stronger evidence is available. The decision to incorporate APCs into a BBG/GTR protocol should be made on an individualised basis, weighing the biological rationale against procedural complexity, cost, and current limitations of the supporting evidence. To establish a definitive clinical standard, future research must shift toward full standardisation of APC processing protocols, expanded database screening frameworks inclusive of gray literature, and large‐scale, methodologically rigorous multicentre RCTs. Furthermore, prospective trials should ideally integrate multivariable stratification to evaluate patient‐level factors such as systemic comorbidities, tobacco consumption profiles, and genetic polymorphisms. Uncovering these complex host‐material interactions is critical to identifying the precise clinical subpopulations that derive the greatest therapeutic benefit from adjunctive APC therapy in periodontal regeneration.

## 5 Conclusion

The findings of this systematic review suggest that the addition of APCs to BBG and GTR for treating intrabony periodontal defects does not yield statistically significant improvements in PPD reduction or CAL gain. However, the available evidence is constrained by the limited number of RCTs, variability in APC preparation protocols and a lack of recent investigations. Furthermore, only one study evaluated PRF, while the remaining four focused on PRP, making it difficult to draw definitive conclusions regarding the overall efficacy of APCs.

To strengthen evidence‐based approaches in periodontal regenerative therapy, future research should adopt standardised protocols, include larger sample sizes and extend follow‐up durations. Additionally, greater emphasis should be placed on exploring the regenerative potential of PRF to better elucidate its role in improving clinical outcomes and to establish clearer conclusions about the adjunctive benefits of APCs.

## Author Contributions

**Robert Chan:** literature searching – preliminary scouting, risk of bias review, writing – primary drafting, manuscript review and editing. **Hugo Lau:** literature searching – preliminary scouting, data curation – data collection, writing – primary drafting, manuscript review and editing. **Michael Liao:** literature searching – formal searching and article exclusions, data curation – data collection, writing – manuscript review and editing. **Jiwoo Park:** literature searching – preliminary scouting, risk of bias review, writing – manuscript review and editing. **Aaron Ting:** literature searching – preliminary scouting, formal searching and article exclusions, data curation – data collection, analysis and visualisation, writing – primary drafting, manuscript review and editing. **Yi Yang Xu:** literature searching – preliminary scouting, formal searching, and article exclusions, data curation – data collection and analysis, writing – primary drafting, manuscript review and editing. **Tulio Fernandez‐Medina:** project administration, framework conceptualisation, manuscript review and editing.

## Funding

The authors have nothing to report.

## Conflicts of Interest

The authors declare no conflicts of interest.

## Supporting information


Supporting File

## Data Availability

The data that support the findings of this study are available from the corresponding author upon reasonable request.
