# Supplementary material for: The Efficacy of Adjunctive Platelet Concentrates in Combined Guided Tissue Regeneration and Bovine Bone Grafting for Intrabony Defects: A Systematic Review
Source: Clin Exp Dent Res. 2026 Jul 26;12(4):e70421. doi: 10.1002/cre2.70421 (PMC13401839; doi:10.1002/cre2.70421)
Supplement: Supplementary file 1 — Supporting File [file CRE2-12-e70421-s001.docx]

**7. Appendices**

**Appendix A**

***Table A1****. Comprehensive Search Strategy used for the Databases*

| Database | Search strings and key words |
| --- | --- |
| Cochrane | ("bovine" OR "natural bone mineral") AND ("autologous platelet concentrates" OR "PRP" OR "PRF" OR "APC") AND ("Guided Tissue Regeneration" OR "GTR" OR "tissue regeneration") AND ("intrabony defect" OR "intra-bony defect") |
| Web of Science | “autologous platelet concentrates” OR “APC” OR “PRP” OR “PRF” OR “Platelet rich” (All Fields) AND "bovine" OR "Bio-Oss" (All Fields) AND "intrabony" OR "intra-bony" OR "intra bony" (All Fields) AND "guided tissue regeneration" OR "periodontal regeneration" OR "GTR" (All Fields) |
| PubMed | ("bovine") AND ("autologous platelet concentrates" OR "PRP" OR "PRF" OR "APC") AND (“bone graft" OR "guided tissue regeneration" OR "GTR”) AND ("intrabony defects" OR "intra-bony defects") |
| Scopus | TITLE-ABS-KEY ((“autologous platelet concentrates" OR "APC" OR "PRP" OR "PRF" OR "Platelet rich") AND "Bovine" AND ("bone graft" OR "guided tissue regeneration" OR "GTR"), AND ("intrabony defects" OR "intra-bony" OR "intrabony")) |
| Ovid MEDLINE | 1. (bovine OR "natural bone mineral")  2. (“autologous platelet concentrate*” OR PRP OR PRF OR APC)  3. ("Guided Tissue Regeneration" OR GTR OR "tissue regeneration")  4. ("intrabony defect*" OR "intra-bony defect*" OR "intra bony")  5.   1 and 2 and 3 and 4 |

**Appendix B**

***Table B1****. Calculations for Cohen’s Kappa statistic*

|  | | Rater 1 (AT) | | Row Marginals |  |
| --- | --- | --- | --- | --- | --- |
|  | | Include | Exclude |  |  |
| Rater 2 (YX) | Include | 5 | 0 | 5 | $rm^{1}$ |
|  | Exclude | 0 | 3 | 3 | $rm^{2}$ |
| Column Marginals | | 5 | 3 | 8 | n |
|  | | $cm^{1}$ | $cm^{2}$ |  |  |

Observed agreement Pr(a): Sum of figures where both raters agreed, divided by total number of articles, where $n=8$.

$$Pr\left( a \right)=\frac{5+3}{n}=\frac{8}{8}=1$$

Probability of expected agreement Pr(e) calculation:

$$\Pr\left( e \right)=\frac{\left( \frac{cm^{1}\times rm^{1}}{n} \right)+\left( \frac{cm^{2}\times rm^{2}}{n} \right)}{n}$$

$$=\frac{\left( \frac{5\times5}{8} \right)+\left( \frac{3\times3}{8} \right)}{8}$$

$$= \frac{\left( \frac{25}{8} \right)+\left( \frac{9}{8} \right)}{8}$$

$$=\frac{34}{64} \approx{0.5}_{(1s.f.)}$$

Cohen’s Kappa Statistic calculation:

$$k=\frac{\Pr\left( a \right)-\Pr\left( e \right)}{1-\Pr\left( e \right)}$$

$$=\frac{1-0.5}{1-0.5}$$

$$=1$$

**Appendix C**

**Figure C1.** Secondary outcome: Mean PPD Reduction at 6 months

**Figure C2.** Secondary outcome: Mean PPD Reduction at 12 months

**Figure C3.** Secondary outcome: Mean PPD Reduction at 24 months
